# Ni-regulated electronic structure and interfacial kinetics in CoCr multi-component alloys enable efficient and durable hydrogen production

**DOI:** 10.1039/d6ra06412d

**Published:** 2026-09-04

**Authors:** Yifan Li, Qihao Zhang, Yuwen Pan, Rongda Zhao, Depeng Zhao, Fufa Wu

**Affiliations:** a School of Materials Science and Engineering, Liaoning University of Technology Jinzhou 121001 P. R. China Rongdazhaoln@126.com; b School of New Energy, Shenyang Institute of Engineering Shenyang Liaoning 110136 P. R. China; c Liaoning Technical University Fuxin Liaoning 123000 P. R. China

## Abstract

The intrinsic electrocatalytic activity of catalysts is strongly correlated with their electronic structure, surface coordination environment, and density of active sites. Multi-component alloys possess tunable chemical compositions and distinctive structural features, which provide abundant regulatory avenues for optimizing catalytic performance. Herein, ternary CoCr multi-component alloys with varied Ni contents, denoted as Ni_*x*_(CoCr)_100−*x*_ (*x* = 30, 40, 50, 60), were fabricated as research objects to systematically investigate how Ni fraction modulates microstructural evolution and electrocatalytic behaviors toward the hydrogen evolution reaction (HER) and oxygen evolution reaction (OER). Comprehensive electrocatalytic evaluations were implemented in both alkaline freshwater and alkaline seawater electrolytes. In 1.0 M KOH alkaline freshwater, the Ni_50_(CoCr) alloy delivers overpotentials of 120 mV for HER and 276.6 mV for OER at a current density of −10 mA cm^−2^ and 10 mA cm^−2^, respectively. To unravel the interfacial reaction kinetics between the electrode and electrolyte, multi-potential Bode plots and Distribution of Relaxation Times (DRT) analyses were adopted. The Bode phase angle declines with elevated applied potential, indicating a continuous reduction in charge transfer resistance. Meanwhile, DRT spectra exhibit only one dominant characteristic peak whose intensity monotonically decreases with increasing overpotential, unambiguously verifying that the interfacial electrocatalysis is governed solely by the charge-transfer step. Considering the abundant, easily accessible reserves of seawater, it serves as an ideal electrolyte for large-scale electrochemical energy conversion and is more consistent with industrial application scenarios compared to pure freshwater. When tested in 1.0 M KOH + seawater mixed electrolyte, the Ni_50_(CoCr) alloy achieves HER and OER overpotentials of 217.3 mV and 314.3 mV at −10 mA cm^−2^ and 10 mA cm^−2^, respectively. Moreover, this alloy maintains stable electrocatalytic performance over a continuous 100 h chronoamperometric test in both alkaline freshwater and alkaline seawater media, demonstrating outstanding long-term durability. This work offers reliable experimental evidence and theoretical guidance for developing low-cost, high-performance, long-lived non-noble electrocatalysts targeted at direct seawater electrolysis, and facilitates the large-scale and sustainable advancement of green hydrogen energy technologies.

## Abstract 2

Vacuum arc‑melted Ni_*x*_(CoCr)_100−*x*_ alloys show excellent HER/OER bifunctional activity in alkaline freshwater/seawater; Ni‑tuned electron redistribution enables stable Ni_50_(CoCr) for low‑cost green hydrogen electrocatalysis.

## Graphical abstract

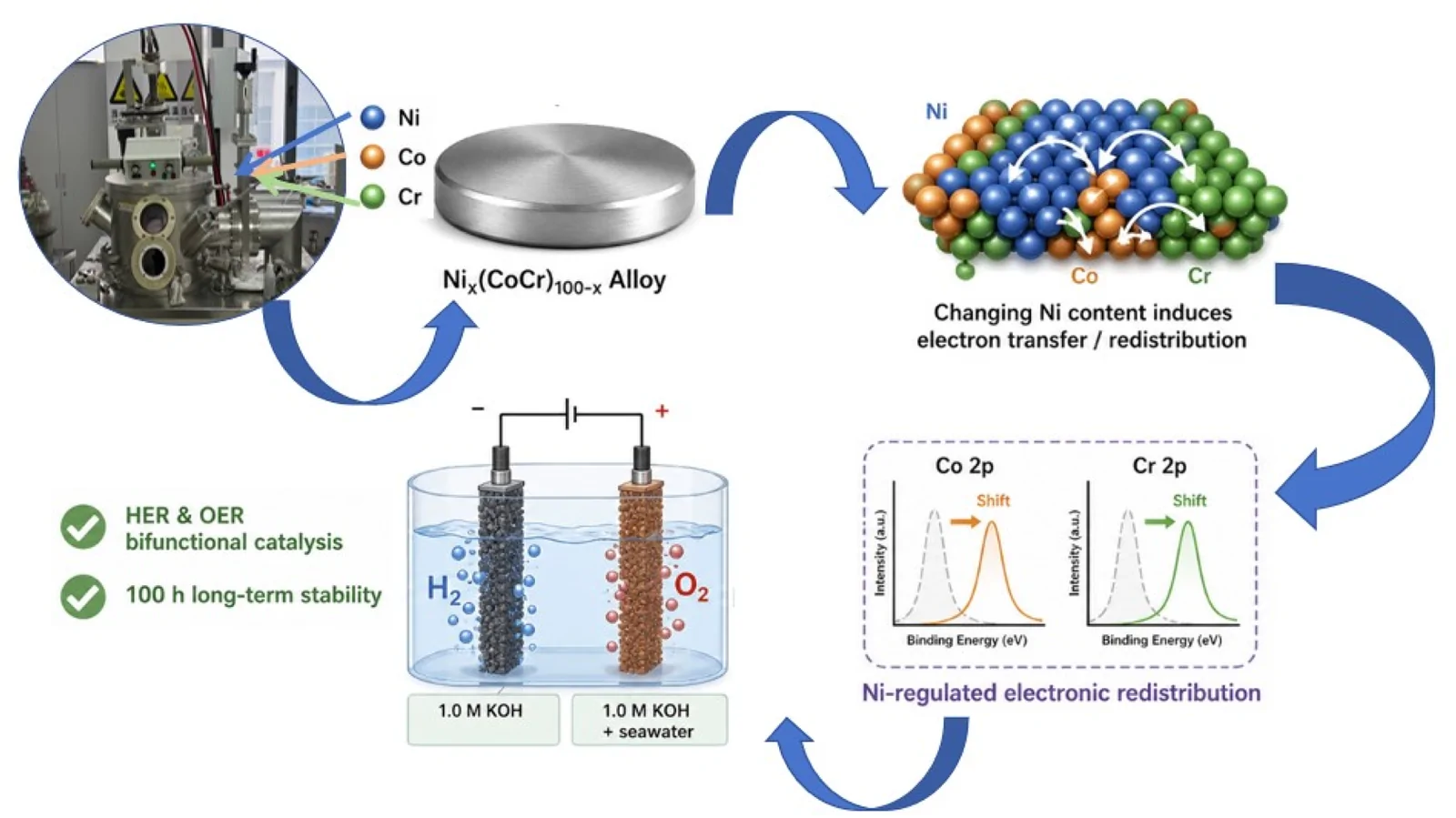

## Introduction

With the intensification of the global energy crisis and the growing severity of environmental issues, developing clean and sustainable new energy technologies has become a core strategic priority for the scientific research and industrial sectors worldwide.^1,2^ Benefiting from its unique merits of high energy density and zero carbon emission, hydrogen energy is recognized as an ideal energy carrier for future energy systems and one of the most promising technical routes to achieve the “Dual Carbon” goals.^3,4^ Water electrolysis converts electrical energy into chemical energy, representing a pivotal approach for large-scale and low-cost green hydrogen production.^5^ Among various water electrolysis technologies, alkaline water electrolysis has gained the widest industrial application owing to its mature technology, low equipment cost and outstanding operational stability.^6,7^ Nevertheless, conventional alkaline electrolysis requires high-purity freshwater as the raw material, which greatly restricts its large-scale deployment amid the global shortage of freshwater resources.^8,9^ Direct electrolysis of abundant natural seawater for hydrogen production can not only break the bottleneck of freshwater supply, but also realize efficient on-site conversion of renewable energy such as wind and solar power in coastal regions, thus possessing profound scientific significance and broad application prospects.^10,11^

Transition metal alloys (*e.g.*, Ni-, Co-, Fe-based materials) have emerged as the preferred research alternatives to noble metal catalysts (Pt, Ir, Ru) due to their abundant reserves, low cost and favorable intrinsic catalytic activity in alkaline media.^12,13^ Among them, Ni-based alloys have attracted extensive research interest for alkaline HER and OER benefiting from their superior electrical conductivity, excellent alkali resistance and promising catalytic capacity.^14,15^ Recent advances in component optimization and interface modulation of Ni-based electrocatalysts for alkaline water splitting have been systematically summarized, providing important guidance for designing high-efficiency non-precious metal catalysts.^16^ For the design of Ni-containing multi-component electrocatalysts, Ni serves as the core active species, and its atomic fraction exerts a decisive influence on catalytic performance. Relevant studies reveal that tuning Ni content can precisely adjust the d-band center of catalysts, thereby modulating the adsorption free energies of hydrogen intermediates (H*) and oxygen-containing intermediates (OH*, O*, OOH*).^17,18^ For instance, the incorporation of Mo into NiMo alloys modulates the electronic configuration of Ni and reduces the hydrogen adsorption free energy, delivering an ultralow HER overpotential of 33 mV at 10 mA cm^−2^.^19^ Significant performance improvement *via* compositional engineering is also observed in ternary NiCo-based alloys. Zhong *et al.* investigated the HER performance of NiCo alloys with varying Ni/Co atomic ratios, and discovered that the alloy with a Ni/Co atomic ratio of 1 exhibits optimal HER activity with an overpotential of 98 mV at 10 mA cm^−2^, outperforming samples with other stoichiometries.^20^ Wang *et al.* reported a ternary NiCoMo alloy that achieves an HER overpotential of merely 45 mV at 10 mA cm^−2^ under alkaline conditions, further verifying the synergistic optimization effect of multiple metal components.^21^ Li *et al.* compared the electrocatalytic behaviors of NiCo and NiCo alloys in simulated seawater, and demonstrated that the NiCoCr alloy delivers an OER overpotential of 287 mV at 10 mA cm^−2^, with less than 10% performance decay after a 100 hour durability test, which greatly surpasses the Cr-free NiCo counterpart.^22^ Collectively, the above literature prove that optimizing Ni content and introducing multi-element synergy are efficient strategies to regulate the electrocatalytic performance of Ni-bearing multi-component alloys.

Alloying modulation *via* introducing secondary Co and ternary Cr elements enables the utilization of intermetallic electronic effects, synergistic effects and lattice distortion effects, so as to precisely optimize the electronic structure, surface active sites and corrosion resistance of catalysts.^23^ Relevant researches demonstrate that the incorporation of Co can effectively adjust the position of the d-band center and optimize the adsorption energies of reaction intermediates (H*, OH*, OOH*), thereby markedly boosting intrinsic catalytic activity.^24^ In contrast, Cr element is expected to facilitate the generation of Cr-rich oxide species which may serve as a potential barrier to alleviate chloride-induced corrosion, as reported in relevant alloy corrosion literature.^25^

To date, extensive investigations have been reported on the electrocatalytic performance of binary Ni–Co and Ni–Cr alloys.^26^ Great efforts have been devoted to exploring advanced electrocatalysts for alkaline and seawater-based water-splitting systems, covering catalyst structural engineering, interfacial electronic modulation and anti-corrosion strategy development.^27^ State-of-the-art studies highlight that rational component tuning and interface optimization are powerful routes to balance catalytic activity and long-term durability under harsh seawater electrolysis conditions.^28,29^ Nevertheless, binary systems usually fail to simultaneously meet the integrated requirements of high activity, high selectivity and ultrahigh stability under complicated seawater environments. In comparison, ternary Ni_*x*_(CoCr)_100−*x*_ alloys possess a wider compositional optimization window *via* continuous tuning of the atomic ratios among Ni, Co and Cr, which endows the materials with the synergistic advantages of multiple active sites. Fine regulation over the Ni content and the relative ratio of Co to promisingly constructs a highly anti-corrosion surface passive structure while maintaining superior catalytic activity, and concurrently suppresses the parasitic chlorine evolution reaction.^30^ However, systematic optimization of Ni content in ternary NiCoCr alloys remains insufficient in existing literature, and an in-depth illustration of the inherent correlation between Ni variation, microstructure and catalytic performance is still lacking.^31,32^ In particular, there is a shortage of thorough analyses on the structure–activity–stability relationship, as well as an insufficient fundamental understanding of their corrosion behaviors and catalytic mechanisms under alkaline conditions.^33^

On this basis, ternary Ni_*x*_(CoCr)_100−*x*_ alloys are selected as the research subject of this thesis to systematically explore the modulation law of Ni content on their crystal structure, surface morphology, electrochemical performance and electrochemical stability. The electrocatalytic activity, reaction kinetics and long-term durability of the alloy series toward hydrogen evolution reaction (HER) and oxygen evolution reaction (OER) are intensively investigated in two electrolytes, namely alkaline freshwater and alkaline natural seawater. Combined with a variety of electrochemical measurements and physicochemical characterizations, the influence of Ni content variation and the synergistic mechanism of Co and Cr dual components are elaborated. In 1.0 M KOH electrolyte, the Ni_50_(CoCr) alloy delivers overpotentials of 120 mV for HER and 276.6 mV for OER at current densities of −10 mA cm^−2^ and 10 mA cm^−2^, with corresponding Tafel slopes of 57.15 mV dec^−1^ and 46.95 mV dec^−1^. Given the abundant reserves of seawater, further electrochemical measurements are carried out in 1.0 M KOH + seawater electrolyte. Seawater electrolysis corresponds to a complicated electrochemical process, where diverse ions migrate and undergo reactions under an electric field, triggering distinct chemical reactions at the anode and cathode. In the seawater-containing alkaline system, the Ni_50_(CoCr) alloy exhibits HER and OER overpotentials of 217.3 mV and 314.3 mV at −10 mA cm^−2^ and 10 mA cm^−2^, with Tafel slopes of 110.81 mV dec^−1^ and 52.5 mV dec^−1^, respectively. This electrocatalyst presents favorable durability in both alkaline freshwater and alkaline seawater environments.

## Results and discussion

### Phase and structure analysis

**Fig. 1:**
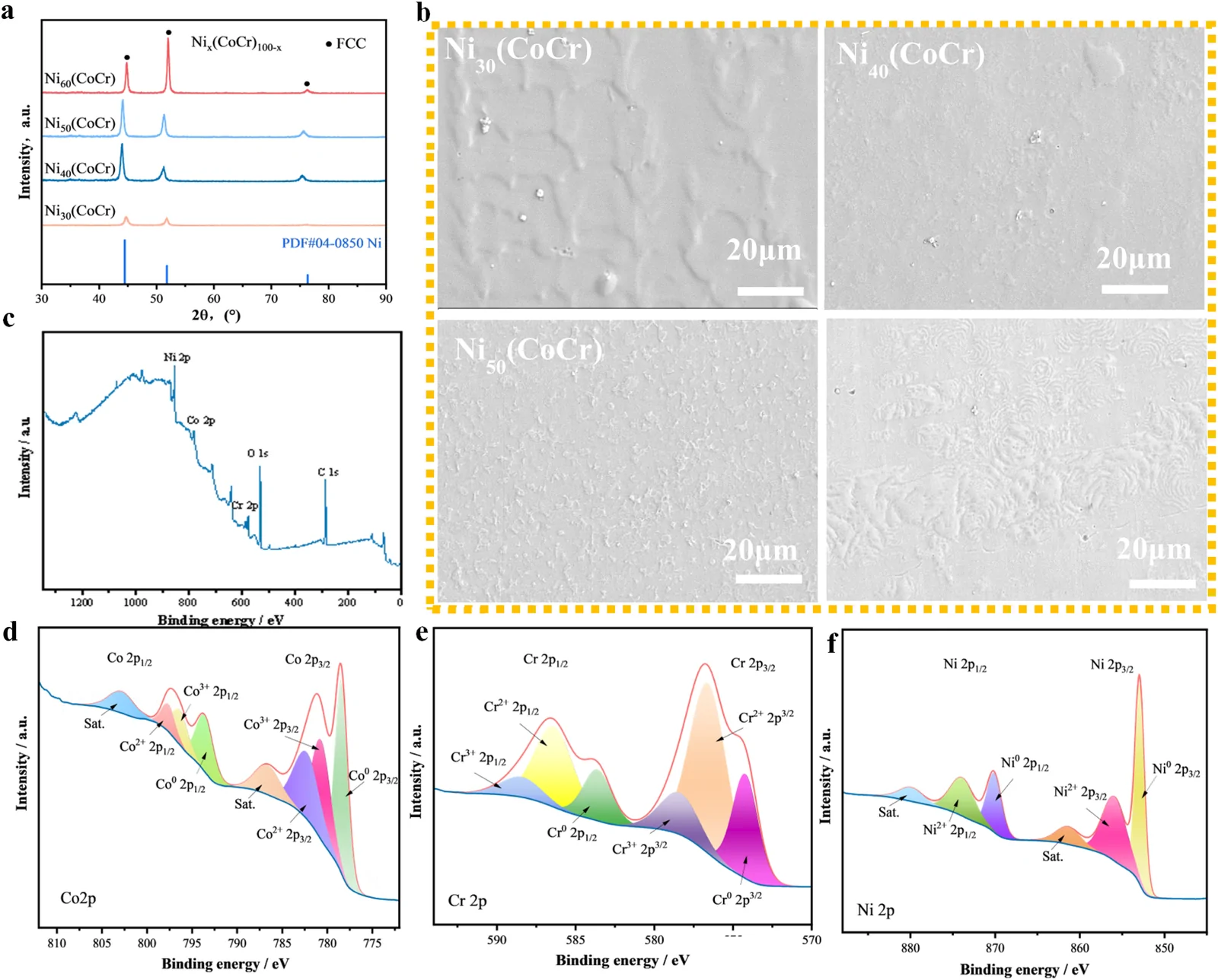
Morphology and structural characteristics of the prepared sample (a) XRD patterns (b) SEM images of Ni_*x*_(CoCr)_100−*x*_ (c) XPS survey (d) XPS of Co 2p (e) Cr 2p (f) Ni 2p.

X-ray diffraction (XRD) measurements were performed on the ternary Ni_*x*_(CoCr)_100−*x*_ (*x* = 30, 40, 50, 60) alloy series, and the corresponding patterns are displayed in Fig. 1a. The standard PDF card (PDF #04-0850) for face-centered-cubic (FCC) Ni is marked in the XRD patterns. All alloy samples within this compositional range only exhibit characteristic diffraction peaks at 44°, 51° and 75°, which can be well indexed to a typical FCC crystal structure. No diffraction signals corresponding to intermetallic compounds or miscellaneous impurity phases are detected in the patterns, verifying the complete solid-solution formation among Ni, Co and Cr elements to generate single-phase FCC solid solutions. Further analysis demonstrates that the intensity and profile of diffraction peaks are highly sensitive to Ni atomic fraction. As the Ni doping ratio increases, the diffraction peaks gradually grow stronger and sharper, indicating continuous improvements in crystallinity and grain regularity.^34,35^ The maximum crystallinity is achieved at a Ni content of 60 at%, accompanied by a denser crystalline matrix and reduced grain boundary defects. Nevertheless, excessively high crystallinity brings certain drawbacks: the limited specific surface area and low defect density would decrease the quantity of catalytic active sites, thus restricting the kinetics of electrochemical reactions.^36,37^ Overall, all as-fabricated Ni_*x*_(CoCr)_100−*x*_ ternary alloys are single-phase FCC solid solutions, whose crystallinity and structural regularity rise with increasing Ni content and reach the optimal state at 60 at% Ni.

### Microstructure and element distribution analysis

Fig. 1b is the result of scanning electron microscope microstructure of Ni_*x*_(CoCr)_100−*x*_ ternary alloy system. The change of Ni content will significantly change the microstructure and compactness of the alloy, and then directly regulate its electrochemical performance. The surface of Ni_30_(CoCr) alloy is undulating, wavy and accompanied by local protrusions. The grain boundary is blurred, the structure is loose, and no continuous grain boundary network is formed. This structure is not only difficult to fully expose the catalytic active sites, but also easy to form corrosion channels at the grain boundaries, which greatly reduces the electrochemical operation stability of the alloy. When the Ni content is increased to 40%, the surface smoothness of the sample is slightly improved, but there are still tiny pores and discrete particles, the grain bonding is poor, and there is still a defect of loose accumulation in some areas. When the Ni content is increased to 50%, the microstructure of the alloy is significantly optimized, the grain size is uniform and the distribution is continuous, and the clear grain boundaries are intertwined to form a dense network equiaxed crystal structure, without coarse grains and pore defects, which can effectively increase the number of active sites and accelerate charge transfer and material transfer rate. When the Ni content increases to 60%, the morphology of the alloy is obviously deteriorated, and a large number of lamellar secondary phases are formed on the surface. The grains grow preferentially and form an uneven laminated structure, which is easy to cause stress concentration and phase separation, accelerate the structural damage in the electrolysis process, and reduce the catalytic stability. In summary, an appropriate increase in Ni content can optimize the microstructure of the alloy, while too high Ni content will cause structural defects. The dense network structure of Ni_50_(CoCr) is the core reason for its excellent electrochemical performance. High-magnification SEM observations for the Ni_*x*_(CoCr)_100−*x*_ alloy series are provided in Fig. S2, further reveal fine microstructural details of each alloy specimen. For Ni_30_(CoCr), blurred grain boundaries and loose, porous micro-regions can be clearly distinguished at high magnification. When the Ni content rises to 40 at%, tiny discrete particles and micro-voids are still observable on the alloy surface. The optimized Ni_50_(CoCr) sample exhibits well-defined interconnected grain-boundary networks and dense equiaxed-grain features without obvious micro-pores. In contrast, abundant lamellar secondary-phase precipitates can be clearly resolved for Ni_60_(CoCr).

In order to verify the distribution uniformity of the main elements in Ni_50_(CoCr) alloy, the EDS element surface distribution was characterized (Fig. S1). The results show that the three main elements of Ni, Co and Cr show a highly uniform distribution in the alloy matrix, and there is no obvious element segregation or local enrichment. The signal intensity of each element is highly consistent with the distribution range, indicating that during the melting process, sufficient mutual dissolution and diffusion occur between the elements, forming a uniform and single alloy matrix, which is consistent with the XRD results. This uniform distribution of elements ensures the uniform distribution of catalytic active sites throughout the surface, thereby achieving an efficient and stable electrocatalytic process.

### Surface chemical composition and valence analysis

In order to explore the chemical valence and electronic structure characteristics of ternary alloy Ni_*x*_(CoCr)_100−*x*_, X-ray photoelectron spectroscopy (XPS) tests were carried out for Ni_50_(CoCr) alloy. The ternary alloy contains three transition metal elements of Ni, Co and Cr, and their valence electrons are arranged in the outer d orbital, which is prone to valence transition, thus providing a large number of active sites for electrocatalytic reactions.^38^ According to the d-band center theory, Ni can strongly adsorb H*, *OH and other reaction intermediates due to its higher d-band center;^39^ Co can regulate the electronic structure of the d band, thereby enhancing the charge transfer efficiency;^40^ Cr can improve the overall corrosion resistance of the material by forming a passivation film.^41^

The test results show that the Ni, Co and Cr elements in the alloy exhibit a typical 2p orbital spin–orbit splitting phenomenon, corresponding to 2p_3/2_ and 2p_1/2_ characteristic peaks, respectively. The XPS full spectrum of Ni_50_(CoCr) ternary alloy is shown in Fig. 1c. The characteristic peaks of Ni 2p, Co 2p, Cr 2p, O 1s and C 1s in the spectrum are clearly distinguishable, indicating that the initial surface chemical state of the alloy is uniform. The XPS spectrum of each element of the alloy can be analyzed. The characteristic peaks of metallic Co^0^ appear at the binding energy of 778 eV and 793 eV in the Co 2p high-resolution XPS spectrum (Fig. 1d), which proves the presence of elemental Co in the sample. At the same time, the characteristic peaks of oxidized Co were observed at 781 eV and 797 eV, corresponding to the 2p_3/2_ and 2p_1/2_ orbital energy levels of Co^3+^ and Co^2+^, and two obvious broad satellite peaks (about 786 eV and 803 eV) appeared in the high binding energy region, which further proved that Co coexisted in multiple valence states.^42,43^ The Cr 2p XPS spectrum (Fig. 1e) consists of two split peaks of 2p_1/2_ and 2p_3/2_, of which the metal Cr^0^ characteristic peak at 574 eV and the oxidation state characteristic peaks of Cr^2+^ and Cr^3+^ in the range of 576–587 eV.^44^ The Ni 2p XPS spectrum (Fig. 1f) shows zero-valent Ni characteristic peaks at 852 eV (Ni^0^ 2p_3/2_) and 870 eV (Ni^0^ 2p_1/2_), corresponding to the 2p_3/2_ and 2p_1/2_ orbital signals of Ni^2+^ at 855 eV and 873 eV, and there are also significant satellite peaks on the high binding energy side.^45^

### Study on electrocatalytic performance

In order to systematically study the electrocatalytic characteristics of ternary alloy Ni_*x*_(CoCr)_100−*x*_ (*x* = 30, 40, 50, 60) in alkaline water electrolysis and alkaline seawater electrolysis environment, this study tested the hydrogen evolution (HER) and oxygen evolution (OER) catalytic activity of samples with different Ni contents in 1.0 M KOH solution and 1.0 M KOH + seawater electrolyte, respectively.

**Fig. 2:**
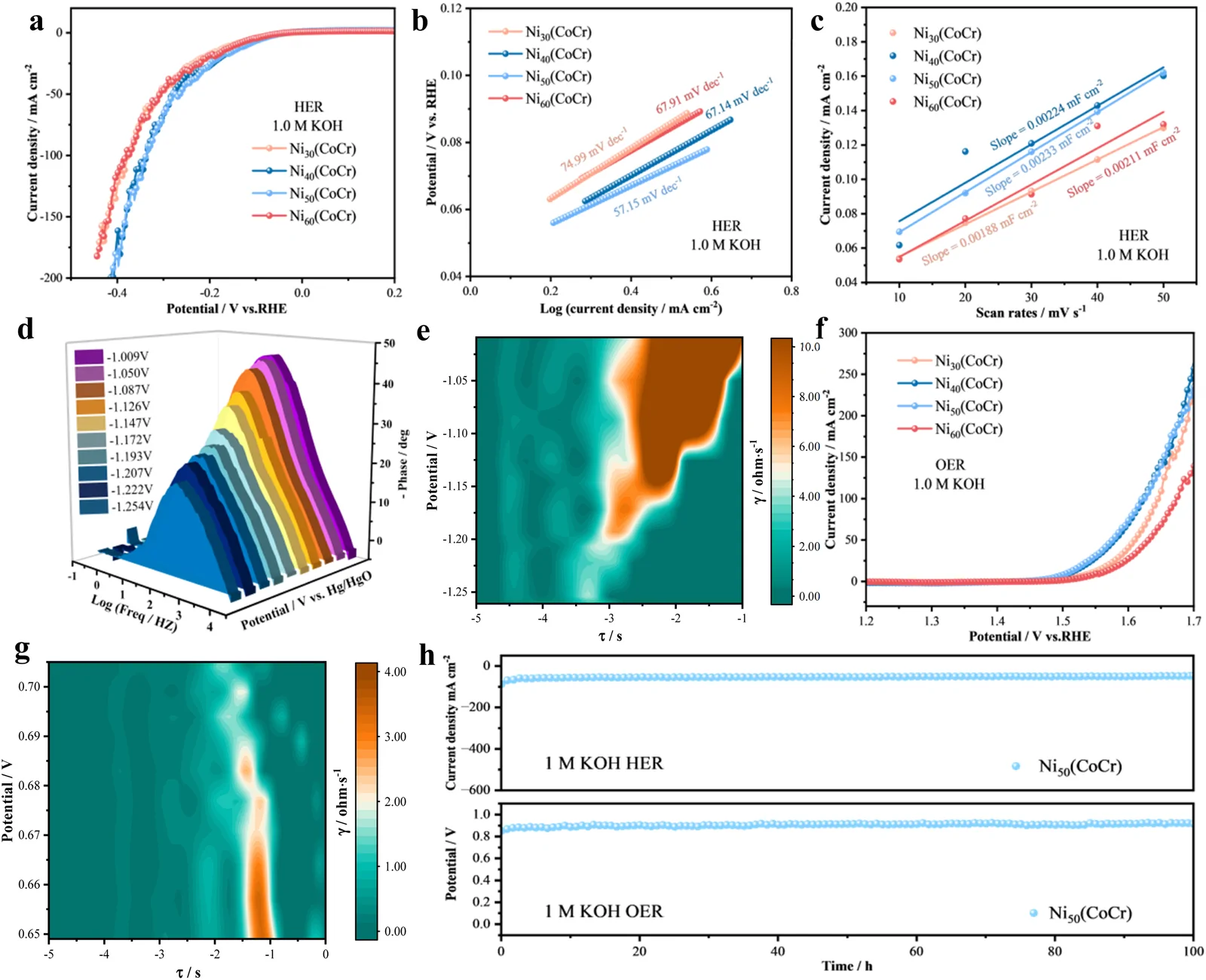
HER/OER performances in 1.0 M KOH solutions (a) polarization curves at scan rate of 5 mV s^−1^ of HER (b) Tafel plots of HER (c) curves of double-layer capacitance of HER (d) Bode plots at multiple voltages of HER (e) DRT spectrum of Ni_50_(CoCr) sample of HER (f) polarization curves at scan rate of 2 mV s^−1^ of OER (g) DRT spectrum of Ni_50_(CoCr) sample of OER (h) stability tests.

The HER performance of the material was tested under a three-electrode system. According to the trend of the linear sweep voltammograms (LSV) curve (Fig. 2a), Ni_50_(CoCr) exhibits the best performance at the same overpotential. At a current density of −10 mA cm^−2^, it is 120 mV, which is less than Ni_30_(CoCr) (149.7 mV), Ni_40_(CoCr) (126.6 mV) and Ni_60_(CoCr) (143.6 mV), meaning that its hydrogen evolution reaction rate is the fastest and the catalytic activity is the best. It is shown that the Ni content has a significant regulatory effect on the hydrogen evolution catalytic activity of the alloy, and the performance comparison of different materials is shown in the histogram (Fig. S3a). In order to explore the reaction kinetics of the material, the LSV curve was further processed to obtain the Tafel slope (Fig. 2b). The Tafel slope of Ni_50_(CoCr) is 57.15 mV dec^−1^, which is smaller than that of other components, indicating that it follows a more favorable Volmer–Heyrovsky mechanism in alkaline media. The energy barrier of proton adsorption and charge transfer is lower, and the kinetic process is more rapid.^46,47^ The excellent HER performance of Ni_50_(CoCr) is essentially derived from the synergistic effect of Ni, Co and Cr: Ni acts as the main active center to provide efficient hydrogen adsorption sites; the addition of Co optimizes the electronic structure of the alloy and reduces the free energy of hydrogen adsorption. Cr further improves the interface charge transfer efficiency by inducing lattice distortion.^48^ Electrochemically active surface area (ECSA) is an important parameter for evaluating electrocatalytic performance. Since ECSA is positively correlated with the electric double layer capacitance *C*_dl_, it can be estimated by *C*_dl_ (Fig. 2c). The *C*_dl_ value of Ni_50_(CoCr) is 0.00233 mF cm^−2^, which is higher than Ni_30_(CoCr) (0.00188 mF cm^−2^), Ni_40_(CoCr) (0.00224 mF cm^−2^) and Ni_60_(CoCr) (0.00211 mF cm^−2^), which proves that it has a larger electrochemically active area. This is consistent with the SEM results.

In order to further analyze the kinetic behavior of the catalyst, we conducted an electrochemical impedance spectroscopy (EIS) test on the sample, (Fig. S3b) is the HER impedance spectrum of the Ni_*x*_(CoCr)_100−*x*_ alloy. The curves of each component are similar, showing good conductivity and fast ion diffusion rate, which provides a guarantee for efficient HER. For Ni_50_(CoCr), the multi-potential impedance test was further carried out. Fig. 2d is the Bode diagram of its phase angle. A wide phase angle peak appeared in the middle and high frequency region, and as the potential shifted negatively, the low frequency phase angle gradually decreased, and the charge transfer resistance continued to decrease, indicating that the reaction was controlled by charge transfer and diffusion, and gradually dominated by charge transfer. This rule is mutually confirmed with the DRT results in Fig. 2e: the DRT peak shape is narrow, the intensity is high, and the relaxation time distribution is concentrated, indicating that the HER process only has a single dominant electrochemical step, no obvious passivation film and diffusion limit, and the electrode interface is stable and efficient.^49^

Similarly, the OER performance was tested in a three-electrode system. The test results showed that the catalytic activity of the ternary alloy was highly consistent with that of HER. The Ni_50_(CoCr) alloy exhibited optimal performance due to surface dynamic reconstruction and multi-element synergistic effect. It can be seen from Fig. 2f that Ni_50_(CoCr) alloy has the lowest OER overpotential of 276.6 mV at a current density of 10 mA cm^−2^, which is lower than that of Ni_30_(CoCr) (313.3 mV), Ni_40_(CoCr) (280.2 mV) and Ni_60_(CoCr) (328.9 mV) alloys (Fig. S3c), which fully proves its catalytic advantage for OER in alkaline freshwater environment. The Tafel curve analysis of Fig. S3d shows that the OER Tafel slope of Ni_50_(CoCr) alloy is 46.95 mV dec^−1^, which is lower than that of other components, indicating that its OER reaction kinetics is faster. The *C*_dl_ results (Fig. S3e) show that the *C*_dl_ value of Ni_50_(CoCr) alloy (0.01042 mF cm^−2^) is much higher than that of other components, indicating that the electrochemical active region of this component alloy is much larger than that of other components, which further illustrates the excellent catalytic performance of Ni_50_(CoCr) alloy. The Nyquist diagram of Ni_*x*_(CoCr)_100−*x*_ alloy is shown in Fig. S3f. The semicircle diameter of Ni_50_(CoCr) alloy is small, and the interface charge transfer resistance is small, which provides a smooth electron transport and ion migration channel for the efficient OER. The Ni_50_(CoCr) alloy with the best catalytic activity was also subjected to multi-impedance test. Fig. S3g is the Bode diagram of the phase angle of the component. It can be seen that with the positive shift of the applied potential, the phase angle in the middle frequency region continues to decrease, and the charge transfer resistance gradually decreases, indicating that the increase of the overpotential can significantly accelerate the interface charge transfer process, and the mass transfer of the material always maintains high efficiency. The DRT spectrum is shown in Fig. 2g, which shows a single and sharp characteristic peak without additional low-frequency peaks, indicating that the OER process is dominated by interfacial charge transfer. There is no obvious product accumulation or passivation film formation on the electrode surface, and the interface stability is excellent.

The cycle stability test of Ni_50_(CoCr) alloy in 1.0 M KOH (Fig. 2h) showed that Ni_50_(CoCr) alloy exhibited good catalytic stability in the stability tests of HER, OER and total water splitting. At the same time, the comparison of LSV curves before and after the stability test (Fig. S3h and i) intuitively revealed its performance decay law. In the 1.0 M KOH alkaline system, the OER stability test results are shown as follows. After 100 h cycle test, the voltage remains stable, and the LSV curve only shows a slight shift after the cycle, reflecting excellent stability. For the HER stability test, the current density is basically maintained at about −40 mV cm^−2^ within 100 h, with only slight fluctuations. The LSV curve after the test is almost coincident with the initial curve, and the overpotential increase is less than 5 mV.

**Fig. 3:**
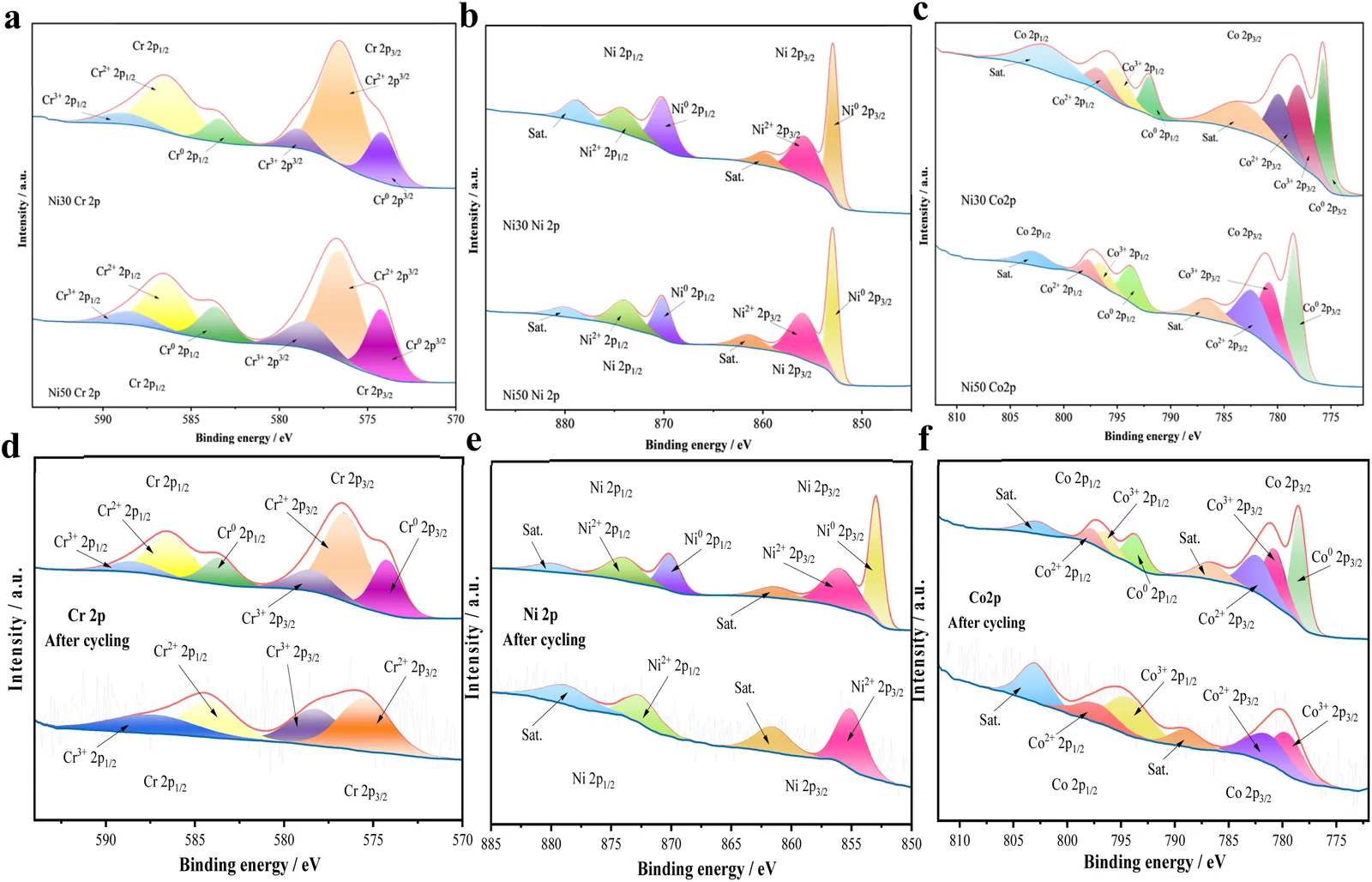
Comparison of XPS images Ni_30_ and Ni_50_ (a) Cr 2p (b) Ni 2p (c) Co 2p before and after cycling (d) Cr 2p (e) Ni 2p (f) Co 2p.

Fig. 3a–c presents the high-resolution Cr 2p, Ni 2p and Co 2p XPS spectra of as-prepared Ni_30_(CoCr) and Ni_50_(CoCr) alloys, aiming to reveal the Ni-modulated electronic-structure effect. For both samples, Cr exists in metallic Cr^0^, Cr^2+^ and Cr^3+^ states; Ni consists of Ni^0^ and Ni^2+^ species; Co is composed of metallic Co^0^, Co^2+^ and Co^3+^, accompanied by characteristic shake-up satellite peaks.

Notably, obvious binding-energy shifts can be observed for Cr 2p and Co 2p core-level peaks when Ni atomic fraction increases from 30 at% to 50 at%. Such peak shifts unambiguously demonstrate interfacial electron redistribution among Ni, Co and Cr, where the variation of Ni content effectively tunes the electronic environment of adjacent Cr and Co sites. Different from the indirect inference merely based on previous literature, this comparison of XPS spectra provides direct experimental evidence for the core viewpoint of “Ni-regulated electronic structure” in this work. The change in electron density around Co and Cr further optimizes the adsorption–desorption behavior of reaction intermediates, which contributes to the enhanced electrocatalytic performance of Ni_50_(CoCr) alloy.

In order to explore the active substances of the reaction, the samples of Ni_50_(CoCr) ternary alloy after IT were analyzed by XPS. Combined with the full spectrum comparison results, it can be found that after the cycle reaction, the characteristic peak intensity of Ni 2p, Co 2p and Cr 2p in the sample is obviously weakened, and the peak shape becomes wider and more dispersed. This phenomenon shows that the long-term electrochemical reaction will cause the surface of the alloy to undergo oxidative reconstruction, and the original metal elements on the surface layer will continue to be converted into various oxides. Combined with the Co 2p fine spectrum (Fig. 3f) after the cycle test, it can be seen that the position and intensity of the orbital characteristic peak of the cobalt element before and after the reaction have changed significantly. The elemental cobalt on the surface of the alloy is basically completely oxidized, all of which are converted into Co^2+^ and Co^3+^, and the two satellite peaks move synchronously to the direction of high binding energy. It can be judged that the cycle process will greatly promote the oxidation reaction of cobalt and provide a large number of reaction sites.^50^Fig. 3d is the 2p orbital fine spectrum of Cr element. The peak of elemental chromium in the sample disappears and completely transforms into Cr^2+^ and Cr^3+^. The characteristic peaks shift to high binding energy as a whole, and the peak area increases significantly.^51^ From the characterization results of Ni 2p fine spectrum (Fig. 3f), the characteristic peak of elemental nickel was greatly weakened after the cyclic reaction, and it was basically completely converted into the oxide of divalent nickel, and the coverage of the satellite peak was also expanded. The oxidation products of nickel can effectively improve the catalytic reaction ability, so that the alloy can maintain good catalytic performance in fresh water environment.^52^ In summary, the cyclic reaction will promote the comprehensive oxidation of metal elements on the surface of the alloy, and gradually generate multivalent oxides of Co, Cr and Ni, so that Ni_50_(CoCr) ternary alloy shows better catalytic ability and reaction kinetics in fresh water system.^53–57^

**Fig. 4:**
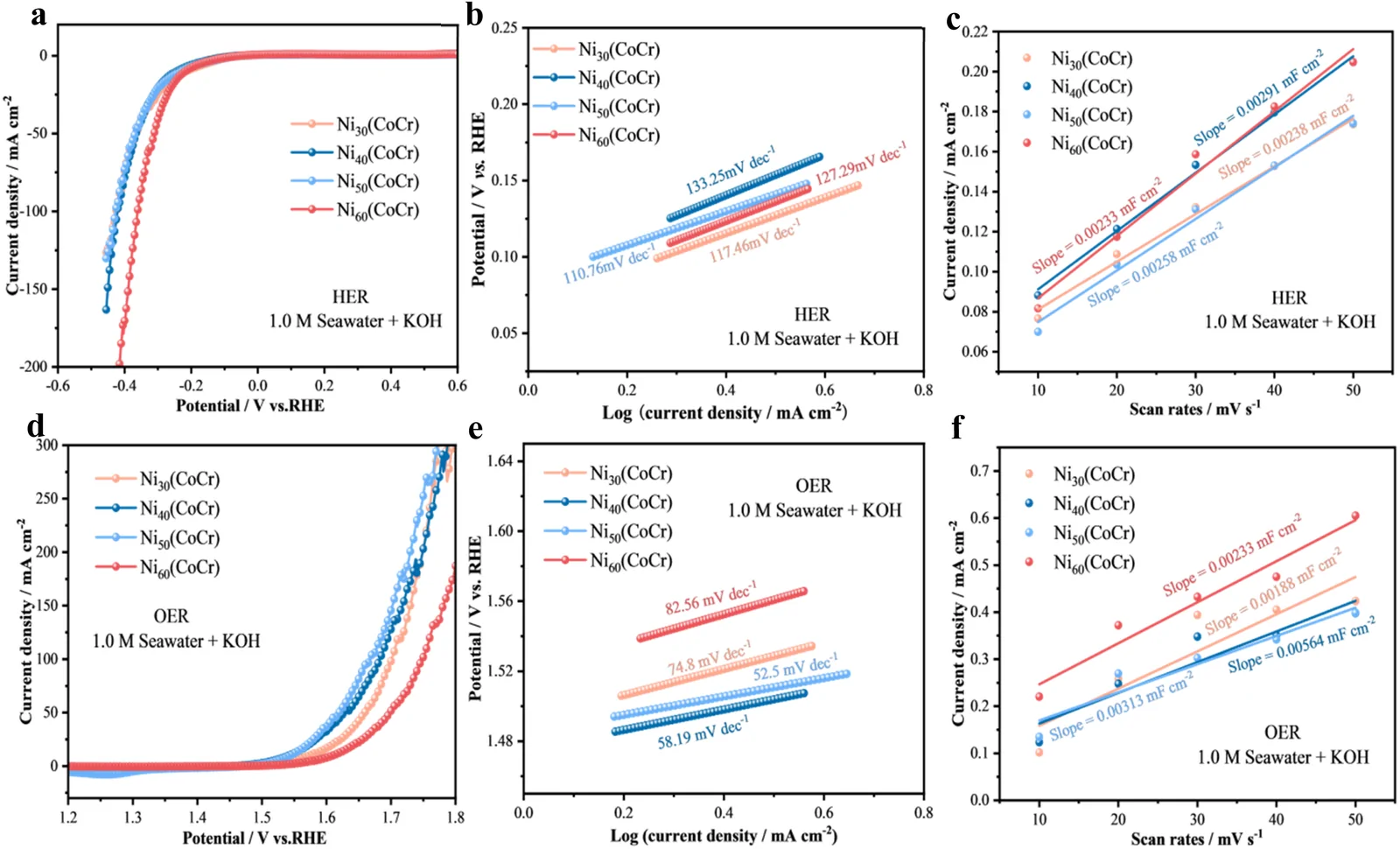
Performances of the electrocatalysts in 1.0 M KOH + seawater (a) polarization curves at scan rate of 5 mV s^−1^ of HER (b) Tafel plots of HER (c) *C*_dl_ of HER (d) polarization curves at scan rate of 2 mV s^−1^ of OER (e) Tafel plots of OER (f) *C*_dl_ of OER.

As a natural water resource with abundant reserves and convenient access, seawater is an ideal medium for large-scale electrochemical energy conversion applications. Compared with pure water system, it is more suitable for practical industrial application scenarios. Therefore, this experiment uses natural seawater instead of pure water to construct an alkaline test system. The material was tested in 1.0 M KOH + seawater, and Ni_50_(CoCr) is still the most excellent component of the alloy system. For its HER, according to the polarization curve (Fig. 4a), at a current density of −10 mA cm^−2^, the overpotential of Ni_50_(CoCr) is 217.3 mV, which is lower than that of Ni_30_(CoCr), Ni_40_(CoCr) and Ni_60_(CoCr). Compared with alkaline freshwater environment, the performance of Ni_50_(CoCr) is slightly decreased, which is inseparable from the complex environment of multi-ion and high corrosion in seawater. It can be seen from the Tafel slope data in Fig. 4b that Ni_50_(CoCr) has more prominent anti-interference performance in seawater environment, and the Tafel slope is only 110.76 mV dec^−1^, which is better than other alloys, proving that it still has efficient and rapid hydrogen evolution reaction kinetics. The excellent performance is based on the optimal atomic ratio of Ni, Co and Cr: the ratio of Ni and Co ensures the intrinsic activity of the catalytic site, and the multi-component synergistic takes into account the corrosion resistance and hydrogen evolution activity to achieve the performance balance of seawater conditions. The *C*_dl_ of the alloy (Fig. 4c) and the EIS impedance spectrum (Fig. S4a) have a small gap between the relevant parameters and the fresh water alkaline system, indicating that the alloy still has a considerable electrochemical active area and fast interface electron conduction in complex seawater, which can effectively avoid chloride ion adsorption corrosion and stabilize interface conductivity. The Bode diagram (Fig. S4b) corresponding to Ni_50_(CoCr) has only one wide characteristic peak and no obvious characteristic peak of passivation film, indicating that the corrosion degree of the electrode is slightly controllable and no thick passivation layer is formed. When the potential is negatively shifted, the low-frequency phase angle continues to decrease, and the charge transfer efficiency continues to increase. It can be seen from the DRT spectrum (Fig. S4c) that the characteristic peaks of the samples are sharp and concentrated, and the relaxation interval is narrow. The hydrogen evolution reaction in seawater environment is still controlled by the charge transfer process, and the catalytic structure of the electrode interface is stable.

The OER performance test was carried out in 1.0 M KOH + seawater, and the performance change rule was highly consistent with that in alkaline freshwater system. From the characterization results of Fig. 4d, it can be seen that Ni_50_(CoCr) alloy still exhibits the best OER catalytic performance in all components, and its anti-interference ability and catalytic environmental adaptability are better than the other alloy components. At a constant current density of 10 mA cm^−2^, the OER overpotential of Ni_50_(CoCr) alloy is 314.3 mV. Under the same test conditions, the overpotentials of Ni_30_(CoCr), Ni_40_(CoCr) and Ni_60_(CoCr) alloys are 355.5 mV, 324.3 mV and 391.3 mV, respectively, which is consistent with the fact that the high concentration of Cl^−^ in the seawater system is prone to chlorine oxidation side reaction (CER) under high potential conditions, and this side reaction forms a competitive reaction with OER. At the same time, a large number of cations such as Na^+^ and Mg^2+^ in seawater are easy to combine with OH on the surface of the electrode to form hydroxide precipitates, covering some catalytic active sites of the electrode, thereby reducing the catalytic reaction efficiency. From the Tafel curve (Fig. 4e) test results, it can be seen that the Tafel slope of the Ni_50_(CoCr) alloy in the seawater system remains 52.5 mV dec^−1^, which is lower than that of the other components of the alloy. It is proved that the component alloy still has the fastest reaction kinetics in the complex ion environment of seawater. This excellent performance advantage is due to the unique synergistic protection and active site retention mechanism of Ni_50_(CoCr) alloy. Under this element ratio, the synergistic effect between Ni and Co effectively maintains the structural stability of the electrode active layer and greatly reduces the erosion damage of the catalytic active site by Cl. Affected by the complex ion composition of seawater and the interference of Cl, the electrochemical double-layer capacitance (Cdl) of Ni_50_(CoCr) alloy is 0.00313 mF cm^−2^ according to the test results (Fig. 4f). The alloy still maintains the catalytic performance advantage far exceeding other components in the seawater system due to the high intrinsic catalytic activity and sufficient active sites endowed by the optimal element ratio. Fig. S4d is the Nyquist impedance diagram of Ni_*x*_(CoCr)_100−*x*_ alloy in alkaline seawater electrolyte. The test results show that the semicircle diameter of Ni_50_(CoCr) alloy impedance spectrum is the smallest, indicating that the charge transfer efficiency of the electrode interface still maintains a high level. The Bode diagram of OER of Ni_50_(CoCr) alloy in alkaline seawater system is shown as (Fig. S4e). As the test potential moves forward, the phase angle in the middle frequency region gradually decreases, the charge transfer resistance continues to decrease, and the peak position of the phase angle slightly shifts to the high frequency region. It is proved that the electrode interface reaction is gradually dominated by charge transfer control, and the reaction rate increases with the increase of overpotential. Fig. S4f is the corresponding DRT map, and the curve only shows a single characteristic peak, and the peak intensity gradually decreases with the positive shift of the potential. This result further confirms that with the increase of overpotential, the charge transfer resistance of the electrode gradually decreases, and the OER catalytic kinetics continues to increase. There is no obvious side reaction and passivation on the electrode surface, indicating that the alloy electrode can maintain a stable and efficient OER catalytic state under the harsh corrosion environment of seawater.

The cycle stability of Ni_50_(CoCr) alloy was further tested in alkaline seawater system. The HER stability test results are shown as (Fig. S4h). In the chronoamperometry current test for 100 h, the current density is stable and there is no obvious attenuation on the whole. After the test, the overpotential is increased by about 10 mV compared with the linear sweep voltammetry curve (Fig. S4g). Fig. S4j is the OER stability test result of this component. During the 100 h chronopotentiometry test, the operating voltage remains stable, and the overpotential of the LSV curve after the cycle increases by 15 mV (Fig. S4i).

To further evaluate the kinetic stability of Ni_*x*_(CoCr)_100−*x*_ alloy catalyst, DRT analysis was performed on the EIS data collected before and after long-term cycling tests under both alkaline and simulated seawater conditions (Fig. S5a–d). Fig. S5a and b correspond to HER and OER DRT comparison in 1 M KOH electrolyte, while Fig. S5c and d exhibit the DRT spectra for HER and OER in 1 M KOH + seawater. The main characteristic peaks related to charge-transfer process maintain nearly identical relaxation time before and after cycling for all test environments, indicating that the rate-determining step of HER and OER remains unchanged after long-time electrolysis. Nevertheless, slight changes in peak intensity can be observed, which is attributed to inevitable surface reconstruction of alloy electrode under harsh alkaline and seawater electrocatalytic conditions. Collectively, the DRT comparison results confirm favorable interfacial kinetic stability of the as-prepared ternary alloy.

**Fig. 5:**
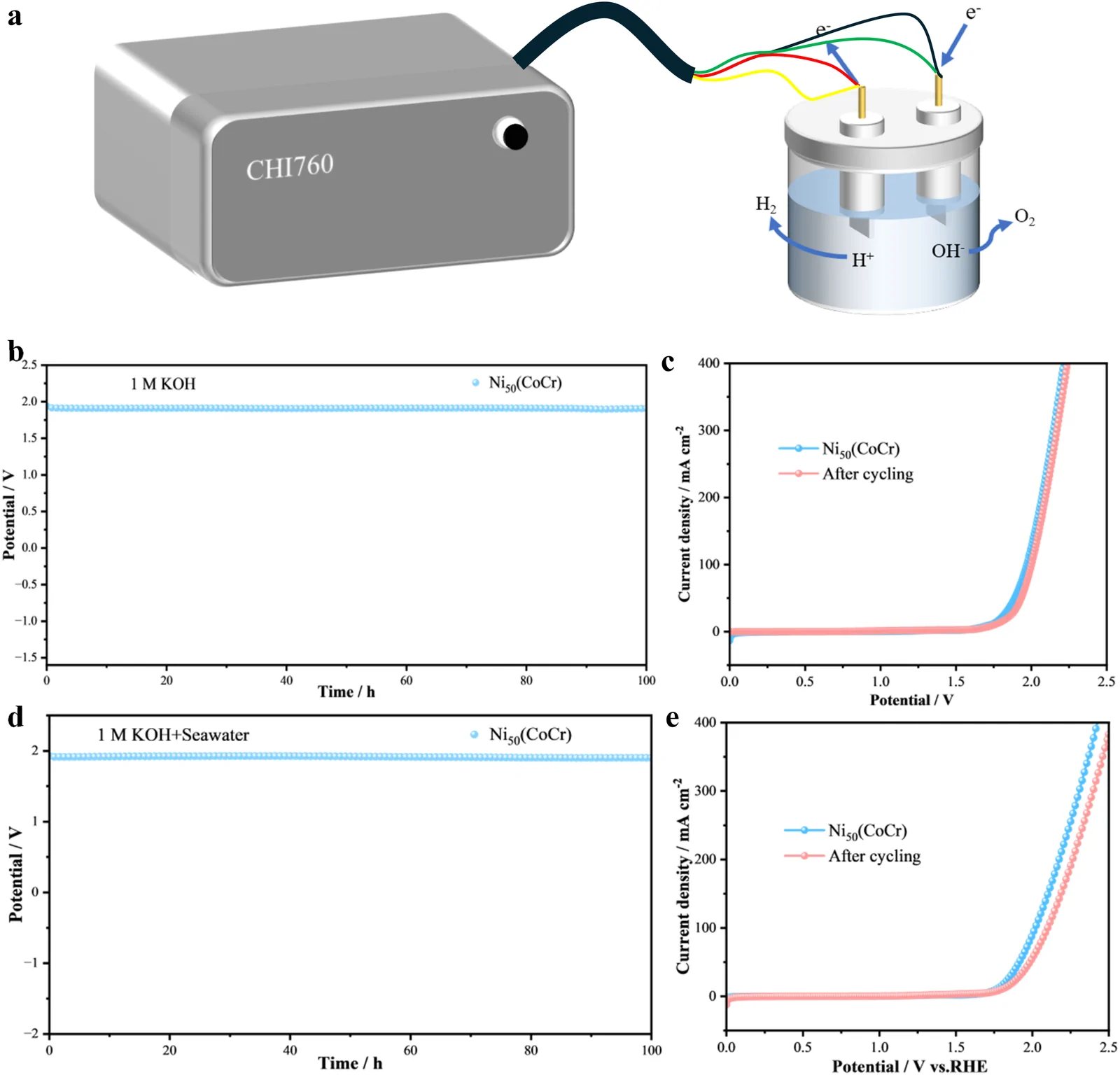
Overall water splitting performance of the electrocatalyst (a) overall hydrolysis performance test tool (b) chronoamperometric stability tests in 1.0 M KOH (c) LSV curves of Ni_50_(CoCr) before and after the cycle in 1.0 M KOH (d) chronoamperometric stability tests in 1.0 M KOH + seawater (e) LSV curves of Ni_50_(CoCr) before and after the cycle in 1.0 M KOH + seawater.

In order to further explore the potential application value of the prepared electrocatalysts in the overall water splitting, the Ni_50_(CoCr) samples were tested for overall water splitting and overall seawater splitting to explore the overall water splitting catalytic activity and long-term operation stability of the Ni_50_(CoCr) ternary alloy. The schematic diagram of the test device is shown in (Fig. 5a), and the long-term service stability is evaluated by the chronoamperometry method (Fig. 5b). After continuous operation for 100 h at a constant working potential, the working potential corresponding to Ni_50_(CoCr) has no obvious attenuation, the curve is straight, and there is no significant fluctuation. It is confirmed that it can stably maintain high-efficiency overall water splitting catalytic performance in a pure water alkaline electrolysis system. Fig. 5c is the LSV polarization curve of Ni_50_(CoCr) before and after 1.0 M KOH stability test. The fresh Ni_50_(CoCr) electrode exhibits excellent bifunctional catalytic activity, and the current response increases rapidly in the high current density range. After a long-term cyclic voltammetry aging test, the polarization curve after the cycle almost completely coincides with the initial curve, and only a slight potential shift occurs at ultra-high current density, which proves that the ternary alloy has excellent cyclic reversibility and structural corrosion resistance in a pure alkaline environment.

In order to simulate the application scenario of industrial seawater electrolysis, the same performance test was carried out in 1.0 M KOH + seawater. The results of chronoamperometry current stability test for up to 100 h (Fig. 5d) further verified the seawater corrosion resistance of Ni_50_(CoCr) ternary alloy. The working potential of Ni_50_(CoCr) ternary alloy remained stable for 100 h in alkaline seawater electrolysis environment without obvious rise or sharp fluctuation, indicating that the Ni–Co–Cr ternary synergistic alloying structure can effectively inhibit the failure behavior of anodic corrosion and active site dissolution caused by chloride ions in seawater, and has long-term catalytic application potential in seawater-based overall water decomposition system (Fig. 5d). The LSV curves of Ni_50_(CoCr) before and after cycling in alkaline seawater electrolysis environment were compared. Due to the influence of corrosive components such as Cl^−^ and impurity ions in seawater, the overall potential at the same current density was slightly higher than that of the pure 1.0 M KOH system. However, the polarization curve after cyclic aging is still highly overlapped with the initial curve, and no obvious catalytic activity attenuation occurs.

Based on the cyclic LSV and long-term chronoamperometry test results of the above dual electrolyte system, Ni_50_(CoCr) ternary alloy has excellent overall water splitting catalytic activity, cyclic durability and seawater corrosion resistance stability. It is a high-performance bifunctional electrocatalytic material suitable for hydrogen production by alkaline electrolysis of pure water and seawater, which makes it a high-efficiency, stable and promising candidate material in water splitting applications.

## Conclusion

In summary, in this study, Ni_*x*_(CoCr)_100−*x*_ series alloy electrodes were successfully prepared by vacuum arc melting process. By adjusting the proportion of Ni element, the microstructure, electronic state, number of active sites and corrosion resistance of the materials can be simultaneously optimized, and an integrated electrocatalytic system with high catalytic activity and strong environmental anti-interference ability can be constructed. The detailed comparison of HER/OER performance between this work and previously reported NiCoCr based metallic electrocatalysts is summarized in Table S1. Binary NiCr and NiCo alloys deliver much higher overpotentials owing to the lack of multi-element synergistic effect. The as-cast Ni–Co–Fe alloy matrix without surface modification also exhibits unsatisfactory bifunctional activity. Although electrodeposited NiCoCr alloy possesses comparable HER performance, its OER overpotential is larger than that of our Ni_50_(CoCr). Such comparison further confirms the superiority of composition-optimized vacuum-arc-melted Ni_50_(CoCr) ternary alloy.

The Ni_50_(CoCr) alloy with the optimal atomic ratio of Ni, Co and Cr can give full play to the synergistic effect of the three elements, simultaneously optimize the electronic structure, enrich the surface active sites and strengthen the corrosion resistance of the matrix, so it shows the optimal electrocatalytic performance in the system. In 1.0 M KOH alkaline freshwater electrolyte, the alloy has a hydrogen evolution overpotential of only 120 mV at a current density of −10 mA cm^−2^, and an oxygen evolution overpotential of 276.6 mV at a current density of 10 mA cm^−2^. Even in the alkaline seawater electrolysis environment with complex ionic components and corrosion risk of chloride ions, Ni_50_(CoCr) still maintains the optimal HER and OER catalytic performance in all components by relying on the surface protection synergistic mechanism constructed by high intrinsic active sites and Cr elements to offset the negative effects of complex electrolytes, which fully proves that precise regulation of the proportion of alloying elements is an effective strategy to overcome the limitations of seawater electrolysis catalysis. At the same time, the long-term stability test results confirm that Ni_50_(CoCr) has excellent cycle durability under both alkaline freshwater and alkaline seawater conditions. The composition control strategy proposed in this study opens up a new path for the design of high-performance non-precious metal electrolytic hydropower catalysts, and also provides important experimental basis and theoretical guidance for the subsequent optimization and industrial application of NiCoCr multi-alloy catalytic system.

## Conflicts of interest

There are no conflicts to declare.

## Supplementary Material

RA-OLF-D6RA06412D-s001

## Data Availability

The data that supports the findings of this study is available from the corresponding authors upon reasonable request. Supplementary information (SI) is available. See DOI: https://doi.org/10.1039/d6ra06412d.

## References

[cit1] Zhao Z., Sui L., Wang Z., Song S., Wang J., Li Y., Zhao D., Li G., Miao L. (2026). Energy Mater..

[cit2] Zhang Q., Zhou H., Zhang B., Ren H., Xu S., Zhao R., Wu F., Miao L. (2026). Fuel.

[cit3] Sazali N. (2020). Int. J. Hydrogen Energy.

[cit4] Wang Z., Tan X., Ye Z., Chen S., Li G., Wang Q., Yuan S. (2024). Green Chem..

[cit5] Zhang W., Yuan W., Zhang X., Ke Y., Wu Y., Bai Y., Jiang S., Tang Y. (2024). J. Mater. Chem. A.

[cit6] Pan Y., Zhao F., Ji G., Xu S., Li R., Zhou H., Zhao R., Zhao D., Wu F. (2025). Int. J. Hydrogen Energy.

[cit7] Ghouri Z. K. , Hussain D. and Hughes D. J., Hydrogen Production from Seawater, Springer Nature, Switzerland, 2024

[cit8] Zhang W., Wei Y., Li J., Xiao H. (2024). Fuel.

[cit9] Umair S. M., Hamdan M. O., Abu-Nabah B. A. (2025). Int. J. Hydrogen Energy.

[cit10] Liu Z., Li H., Yang C., Jiang M., Zhang J., Fu C. (2024). Small.

[cit11] Xin Y., Hua Q., Li C., Zhu H., Gao L., Ren X., Yang P., Liu A. (2024). J. Mater. Chem. A.

[cit12] Zhang R., Xie A., Cheng L., Bai Z., Tang Y., Wan P. (2023). Chem. Commun..

[cit13] Li X., Xu S., Li J., Zhang S., Zhang B., Zhao R., Zhao D., Wu F. (2025). Nanoscale Adv..

[cit14] Mir R. A., Kirk D. W., Thorpe S. J. (2024). J. Alloys Compd..

[cit15] Yang F., Tian X., Luo W., Feng L. (2023). Coord. Chem. Rev..

[cit16] Sun Y., Yang F., Sun S., Wei K., Wang Y., Ma G., An J., Yuan J., Zhao M., Liu J., Liu H., Li Y. (2025). J. Colloid Interface Sci..

[cit17] Xiong L., Wang B., Cai H., Hao H., Li J., Yang T., Yang S. (2021). Appl. Catal., B.

[cit18] Xiao Z., Zhou W., Yang B., Liao C., Kang Q., Chen G., Liu M., Liu X., Ma R., Zhang N. (2023). Nano Mater. Sci..

[cit19] Phan T. P. A. , PhD thesis, Technical University of Denmark, 2025

[cit20] Zhong S., Li J., Cui Z., Tian G., Zhao F., Zhou Z., Jiao H., Liu D., Xiong J., Wang L., Xiang J., Wu F., Zhao R. (2024). J. Mater. Res. Technol..

[cit21] Wang S., Xu B., Huo W., Feng H., Zhou X., Fang F., Xie Z., Shang J., Jiang J. (2022). Appl. Catal., B.

[cit22] Wei R., Zhang K., Zhao P., An Y., Tang C., Chen C., Li X., Ma X., Ma Y., Hao X. (2021). Appl. Surf. Sci..

[cit23] Dong J., Liu X., Lv N., Li H., Li T., Guo Z., Luo J. (2024). J. Alloys Compd..

[cit24] Liu Z., Zhang X., Song H., Yang Y., Zheng Y., Gao B., Fu J., Chu P. K., Huo K. (2020). ChemCatChem.

[cit25] Zhang B., Zhao Y., Li X., Zhou H., Zhao X., Zhao R., Wu F. (2025). RSC Adv..

[cit26] Zhang Z., Ter-Ovanessian B., Marcelin S., Normand B. (2020). Electrochim. Acta.

[cit27] Wang C., Wu X., Sun H., Xu Z., Xu C., Wang X., Li M., Wang Y., Tang Y., Jiang J., Sun K., Fu G. (2025). Energy Environ. Sci..

[cit28] Zhu Y., Wang Y., Zhou Y., Chen M., Wang X., Li M., Wang Y., Tang Y., Li H., Wei L., Fu G. (2026). Angew. Chem., Int. Ed..

[cit29] Li M., Dong W., Zhang X., Xu L., Gao X., Yang Y., Tang Y., Wang Y., Chen L., Fu G. (2025). Adv. Funct. Mater..

[cit30] Ni Q., Zhu M., Yuan Y., Guo S. (2025). J. Mater. Eng. Perform..

[cit31] Zhou H., Xu S., Zhang B., Li X., Lv Z., Zhao D., Zhao R. (2026). Inorg. Chem. Front..

[cit32] Ahmad S., Egilmez M., Abuzaid W., Mustafa F., Kannan A. M., Alnaser A. S. (2024). Int. J. Hydrogen Energy.

[cit33] Dong Y., Zhang Y., Yu J., Qin S., Zhang H., Yang Z., Hu T., Zhang X., Ren Z. (2025). J. Alloys Compd..

[cit34] Tong Y., Jin K., Bei H., Ko J. Y. P., Pagan D. C., Zhang Y., Zhang F. (2018). Mater. Des..

[cit35] Lu Y., Huang C., Fang T. (2023). ECS Meeting Abstracts.

[cit36] Kardes G., Röse P., Wildersinn L., Jeschull F., Korneychuk S., Pundt A., Grunwaldt J. D. (2025). ACS Catal..

[cit37] She C., Hong S., Song N., Zhao Z., Li J., Niu Y., Li C., Dong H. (2024). Inorg. Chem..

[cit38] Wu T., Li X., Li Y., Zhang S., Chang W., Chi J., Wang L. (2025). Chem. Eng. J..

[cit39] Li S., Yue F., Tan H., Zeng K., Fan W., Gong K., Wu X., Dai C., Chen W., Tan X., Smith S. C., Li Y. (2025). Nano Lett..

[cit40] Qian S., Wei J., Wang J., Zhang Y., Xue H., Jiang T., Tian J. (2025). Chin. Chem. Lett..

[cit41] Hong H., Li J., Li S., Lei L., Lin Y., Zhu M., Zhuang L., Xu Z. (2025). Chem. Eng. Sci..

[cit42] Nie Z., Chen M., Zhang L., Feng Q., Hu J., Huang X., Zhou C., Zhou Y., Wågberg T., Hu G. (2023). Chem. Eng. J..

[cit43] Diao F., Kraglund M. R., Cao H., Yan X., Liu P., Engelbrekt C., Xiao X. (2023). J. Energy Chem..

[cit44] Zhang S., Lu Q., Zhang H., Liang K., Tan Y., Sun C. (2020). Int. J. Electrochem. Sci..

[cit45] Guzman-Bucio D. M., Gomez-Sosa G., Cabrera-German D., Torres-Ochoa J. A., Bravo-Sanchez M., Cortazar-Martinez O., Carmona-Carmona A. J., Herrera-Gomez A. (2023). J. Electron Spectrosc. Relat. Phenom..

[cit46] Ren H., Zhou H., Zhang Q., Su Z., Liu H., Zhao R., Zhao D., Zheng Z., Miao L. (2026). J. Colloid Interface Sci..

[cit47] Kucernak A. R., Zalitis C. (2016). J. Phys. Chem. C.

[cit48] Silveira A. F., Urbano J. V. S., Costa J. D., Oliveira J. A. M., Campos A. R. N., de Santana R. A. C. (2025). Electrocatalysis.

[cit49] Danzer M. A. (2019). Batteries.

[cit50] Zhou H., Li X., Zhang B., Ren H., Zhao D., Zhao R., Miao L. (2025). ACS Appl. Mater. Interfaces.

[cit51] Yang P., Jiang Z., Shi Y., Ren X., Liang L., Shao Q., Zhu K. (2023). J. Alloys Compd..

[cit52] Li R., Xu S., Ai Z., Qi J., Wu F., Zhao R., Zhao D. (2025). Int. J. Hydrogen Energy.

[cit53] Tourneur J., Lagrost C., Fabre B. (2023). Advanced Energy and Sustainability Research.

[cit54] Narayanan B., Dhanabal V. M. H., Kumaresan L., Gurusamy S. (2025). Energy Fuels.

[cit55] Arasan N., Akkurt F. (2025). ACS Omega.

[cit56] Darband G. B., Aliofkhazraei M., Rouhaghdam A. S. (2019). J. Colloid Interface Sci..

[cit57] Chen Y., Yang L., Li C., Wu Y., Lv X., Wang H., Qu J. (2024). Energy Environ. Mater..

