# Supplementary material for: Ni-regulated electronic structure and interfacial kinetics in CoCr multi-component alloys enable efficient and durable hydrogen production
Source: RSC Adv. 2026 Sep 4;16(43):52971–82. doi: 10.1039/d6ra06412d (PMC13543213; doi:10.1039/d6ra06412d)
Supplement: Supplementary file 1 [file RA-016-D6RA06412D-s001.pdf]

Supplementary Information (SI) for RSC Advances.
This journal is © The Royal Society of Chemistry 2026

Ni-Regulated Electronic Structure and Interfacial Kinetics in CoCr Multi-Component Alloys Enable Efficient and Durable Hydrogen Production

Yifan Li[a], Qihao Zhang[a], Yuwen Pan[a], Rongda Zhao[a*], Depeng Zhao[b*], Fufa Wu[c*]

aSchool of Materials Science and Engineering, Liaoning University of Technology,

Jinzhou, 121001, P. R. China

bSchool of New Energy, Shenyang Institute of Engineering, Shenyang, Liaoning,

110136, P. R. China

cLiaoning Technical University, Fuxin, Liaoning, 123000, P. R. China

1.Experimental methods:

1.1 Experimental materials:

Commercially available high-purity Ni granules, Co granules and Cr granules (purity ≥ 99.99%) were used as starting raw materials. Potassium hydroxide (KOH, analytical grade, purchased from Aladdin Reagent Co., Ltd.), and natural seawater collected from offshore Huludao, China were also utilized in this work.

1.2 Preparation of block electrode:

According to the designed alloy compositions with varying Ni atomic fractions, the required mass of each metallic raw material was calculated on an atomic percentage basis. An analytical balance with a precision of 0.1 mg was adopted for weighing, and the mass deviation of each metal was controlled within ±2.0 mg. After weighing, the metallic feedstocks were sealed in vacuum sample bags to avoid oxidation upon exposure to air during storage, which would alter their mass and

material performance. Four batches of raw materials corresponding to $Ni_x(CoCr)_{100-x}$ (x=30, 40, 50, 60) were prepared as designed. Subsequently, a DXL-500II non-consumable tungsten inert gas arc melting furnace was used to melt the pure metals under an argon protective atmosphere. Each alloy ingot was remelted four times with a 5 min single melting duration to guarantee compositional homogeneity. Two cycles of magnetic stirring were further implemented to eliminate compositional segregation, uneven temperature distribution and trapped inclusions, thereby ensuring uniform chemical composition of the as-melted ingots. After melting, the surface oxide scale of each ingot was removed via grinding, followed by wire electrical discharge machining (WEDM) to cut multiple specimens with dimensions of 0.25 cm × 0.25 cm. The cut samples were ultrasonically cleaned in petroleum ether and ethanol sequentially and then dried. One surface of each specimen was ground stepwise using metallographic abrasive papers ranging from 400 to 1500 grit, followed by final polishing with polishing paste. The obtained as-cast $Ni_x(CoCr)_{100-x}$ (x=30,40,50,60) specimens were reserved for subsequent characterizations and electrochemical tests.

1.3 Material properties test:

The crystal structures of as-cast $Ni_x(CoCr)_{100-x}$ (x = 30, 40, 50, 60) specimens were characterized by an X-ray diffractometer (Model D/MAX2500) with Cu Kα radiation. The measurement parameters were set as tube current of 100 mA, tube voltage of 40 kV, scanning 2θ range of 20°-100°, and scanning rate of 8° min^{-1}. X-ray photoelectron spectroscopy (XPS, ESCALAB250) was utilized to analyze the phase composition, elemental distribution and surface oxidation states of the alloys. A

Zeiss-Sigma 500 scanning electron microscope (SEM) was adopted to observe the surface morphology and microstructural features of all samples.

1.4 Electrochemical test:

Electrochemical measurements were carried out on a CHI760E electrochemical workstation using a standard three-electrode configuration. The electrochemical behaviors of the samples were tested in two electrolytes: 1.0 M KOH aqueous solution (pH = 13.7) and mixed electrolyte consisting of 1.0 M KOH+seawater (pH = 13.51). A Hg/HgO electrode served as the reference electrode, platinum foil and carbon rod were used as the counter electrodes, and the as-prepared alloy specimens acted as the working electrode. Prior to formal measurements, the working electrode was activated via 40 cyclic voltammetry (CV) cycles at a scan rate of 500 mV s^{-1} to saturate the electrolyte with reaction gases and stabilize the catalytic surface. All measured potentials were calibrated to the reversible hydrogen electrode (RHE). A series of electrochemical characterizations including linear sweep voltammetry (LSV), cyclic voltammetry (CV), electrochemical impedance spectroscopy (EIS) and chronoamperometry (i-t) were recorded. The potential values for HER and OER were converted to the RHE scale based on the following formula: E(Vs RHE)=E(Vs Hg/HgO)+0.059*pH+0.098 V.

2.Supplementary Figure：

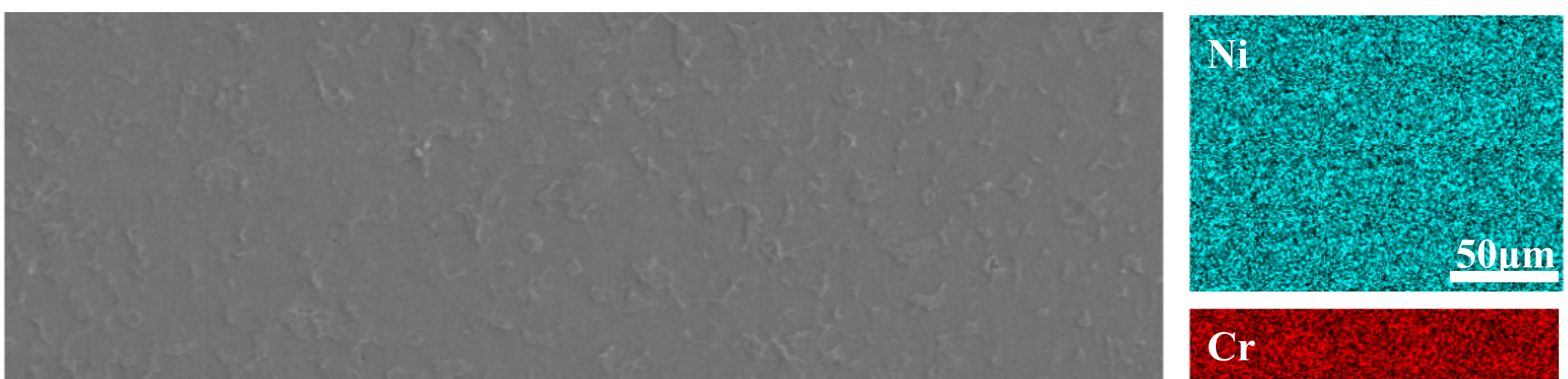

FigureS1. Elemental mapping of Ni_{50}(CoCr) alloys

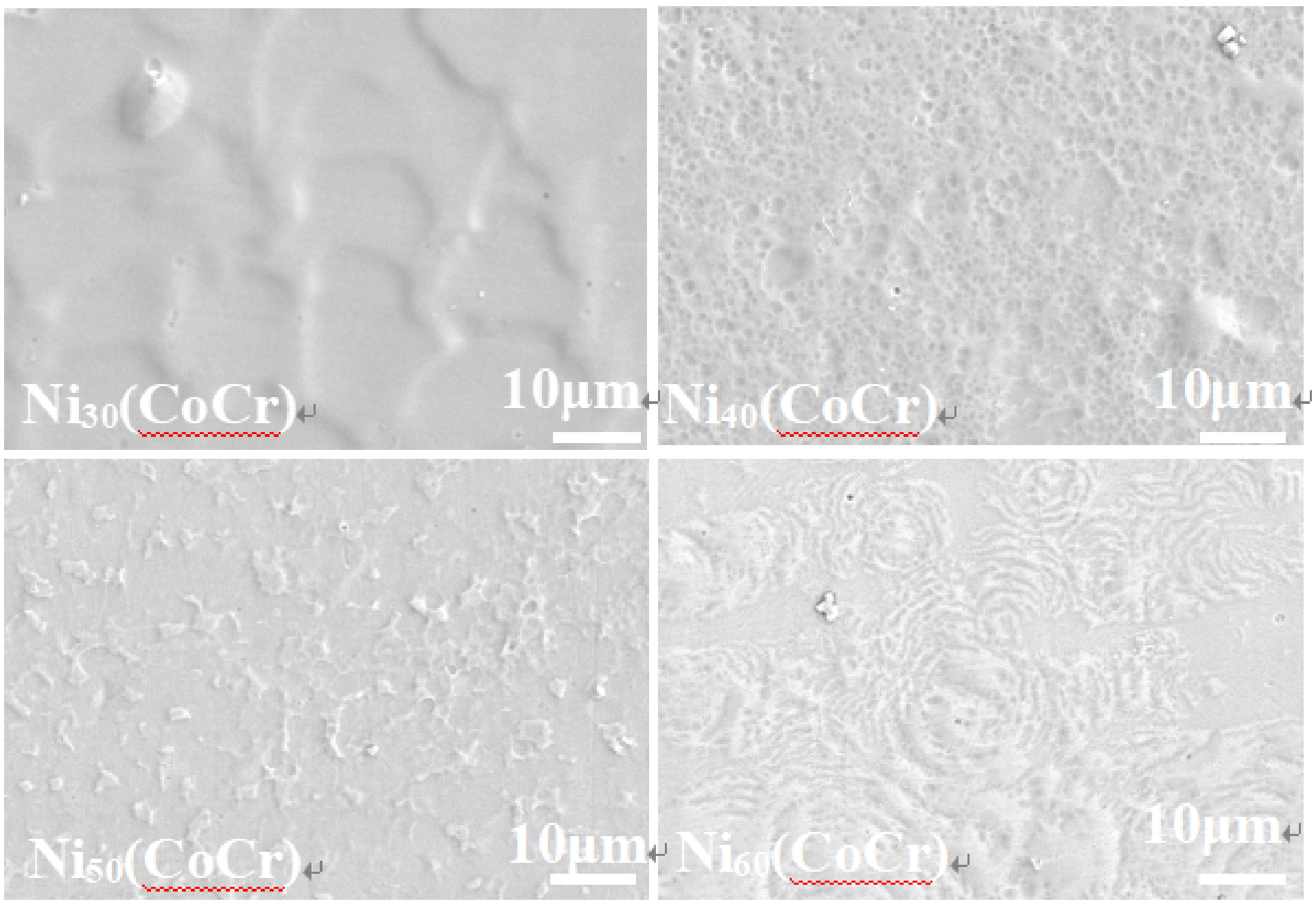

FigureS2. High-magnification SEM observations for the $Ni_x(CoCr)_{100-x}$ alloy

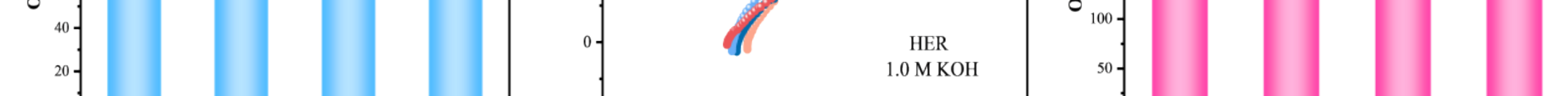

FigureS3. Performances in 1.0 M KOH solutions (a) Overpotential at current density of -10 mA cm^{-2} of HER (b) Nyquist plots of HER (c) Overpotential at current density of -10 mA cm^{-2} of OER (d) Tafel plots of OER (e) Curves of double-layer capacitance of OER (f) Nyquist plots of OER (g) Bode plots at multiple voltages of OER (h) LSV Curves of Ni_{50}(CoCr) before and after the cycle in 1.0 M KOH of OER (i) LSV Curves of Ni_{50}(CoCr) before and after the cycle in 1.0 M KOH of HER

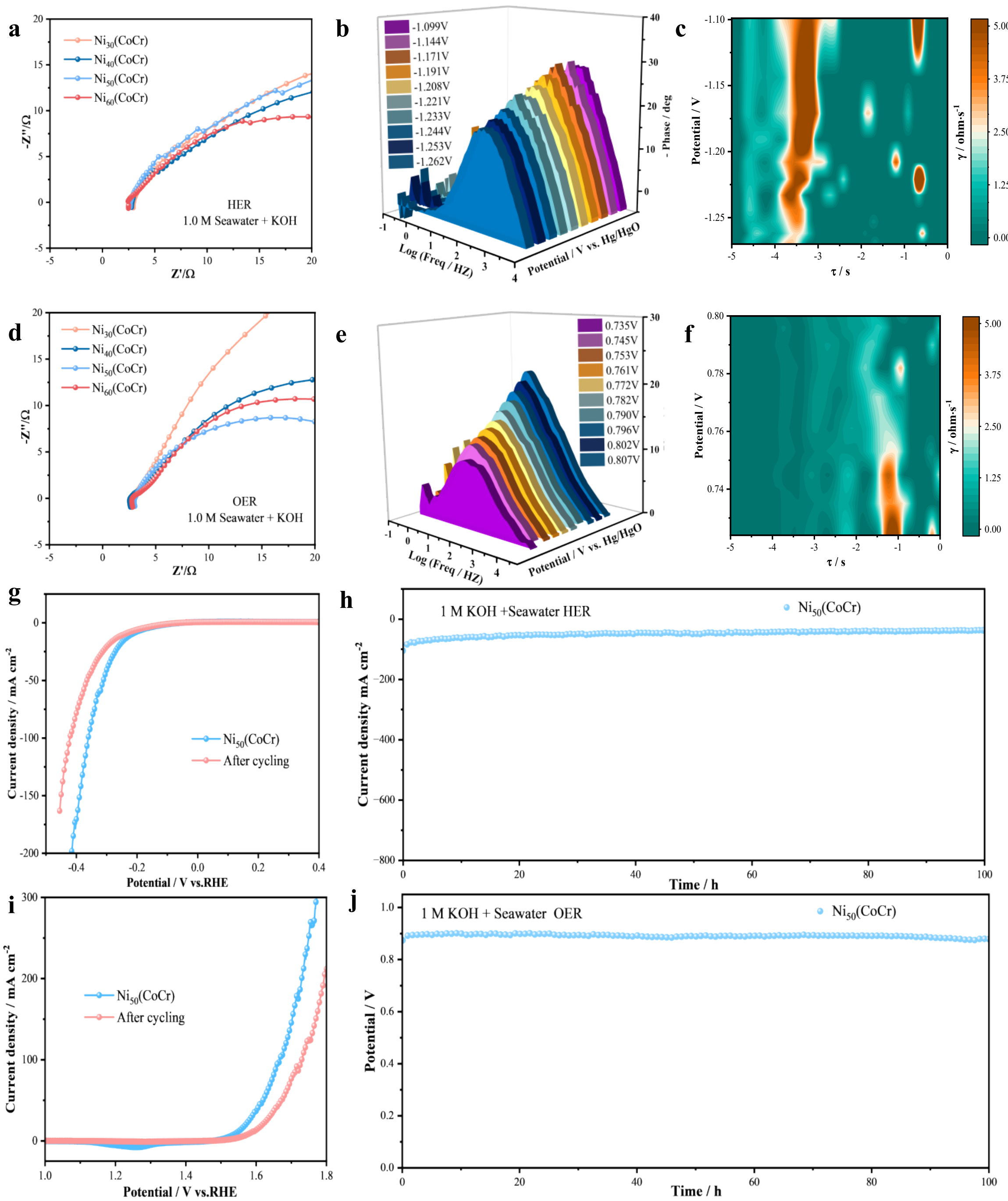

FigureS4. Performances of the electrocatalysts in 1.0 MKOH+seawater (a) Nyquist plots of HER (b) Bode plots at multiple voltages of HER (c) DRT spectrum of $Ni_{50}(CoCr)$ sample of HER (d) Nyquist plots of OER (e) Bode plots at multiple voltages of OER (f) DRT spectrum of $Ni_{50}(CoCr)$ sample of OER (g) LSV Curves of $Ni_{50}(CoCr)$ before and after the cycle in 1.0 M KOH of HER (h) Chronoamperometric stability tests in alkaline seawater of HER (i) LSV Curves of $Ni_{50}(CoCr)$ before and after the cycle in 1.0 M KOH of OER (j) Chronopotentiometry stability tests in 1.0 MKOH+seawater of OER

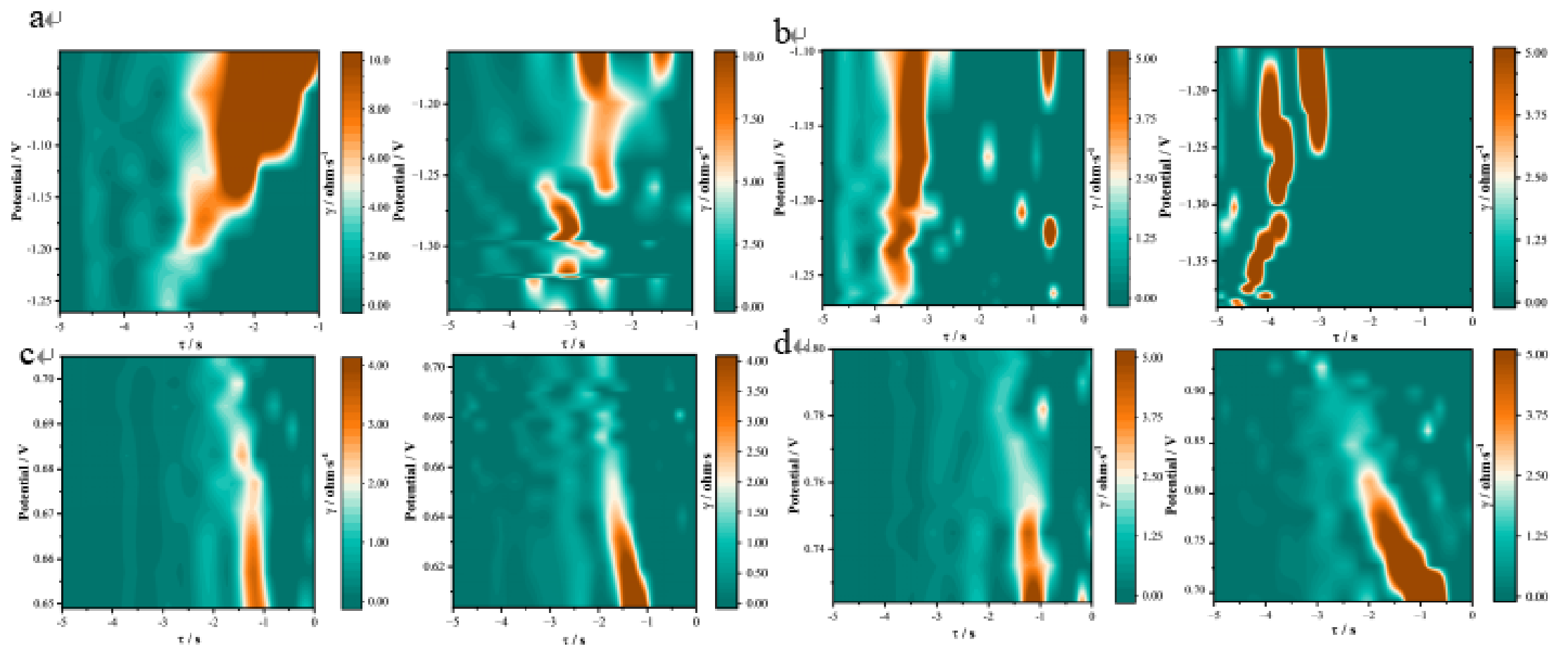

Figure S5.DRT plots derived from EIS before and after stability cycling. (a) HER in 1 M KOH; (b) OER in 1 M KOH; (c) HER in 1 M KOH+seawater; (d) OER in 1 M KOH+seawater

Catalyst	Electrolyte	η_{10} HER (mV)	η_{10} OER (mV)	Ref.
This work Ni_{50}(CoCr)	1 M KOH	120	276.6	-
Electrodeposited NiCoCr alloy	1 M KOH	110	292	[53]
Binary Ni-Cr alloy	1 M KOH	314	327	[54]
Binary Ni-Co alloy	1 M KOH	242	335	[55]
NiFeCo	1 M KOH	91	316	[56]
As-cast Ni-Co-Fe alloy matrix	1 M KOH	148	312	[57]

Table S1. Comparison of catalytic performance for NiCoCr-related alloy electrocatalysts in 1 M KOH
